# Effects of Ionizing Radiation on the Biophysical Properties of Type I Collagen Fibrils

**DOI:** 10.1371/journal.pone.0319777

**Published:** 2025-04-02

**Authors:** Kester Ng, Nader Allam, Mehrnoosh Neshatian, Mina Vaez, Liisa M. Hirvonen, Ernest Lam, Alex Vitkin, Laurent Bozec

**Affiliations:** 1 Faculty of Dentistry, University of Toronto, Toronto, Canada,; 2 Department of Medical Biophysics, University of Toronto, Toronto, Canada,; 3 Centre for Microscopy, Characterisation & Analysis, The University of Western Australia, Perth, Australia; University of South Carolina, UNITED STATES OF AMERICA

## Abstract

Ionizing radiation is extensively employed in both diagnostic and therapeutic medical practices. The impact of this radiation on collagen, a primary structural protein in humans, remains underexplored, particularly at varying doses and hydration states. This study explores the impact of ionizing radiation on type I collagen fibrils at three radiation doses (diagnostic, therapeutic, and sterilization) and under two hydration conditions using an engineered acellular collagen membrane to reflect varying biological conditions. Techniques including atomic force microscopy (AFM), fluorescence lifetime imaging microscopy (FLIM), and Attenuated total reflectance-Fourier transform infrared spectroscopy (ATR-FTIR) were utilized to assess changes in mechanical properties, biochemical stability, and molecular structure respectively. Our results demonstrate that ionizing radiation alters the mechanical properties of collagen fibrils, notably indentation modulus, which reflects changes in stiffness or elasticity. These modifications depended on the hydration state at the time of radiation exposure; hydrated fibrils typically exhibited increased stiffness, suggesting enhanced cross-linking, whereas dehydrated fibrils showed reduced stiffness, indicative of structural weakening, possibly due to bond breakdown. Morphological changes were minimal, suggesting that radiation primarily affects the internal structure rather than the overall appearance of the fibrils. Biochemically, variations in fluorescence lifetimes highlighted changes in the collagen’s biochemical environment, dependent on the dose and hydration state. Despite these biochemical and mechanical changes, FTIR analysis indicated that the primary structure of collagen was largely preserved post-irradiation for all examined dose levels. These findings imply that radiation can modify the mechanical properties of collagen, potentially affecting tissue integrity in clinical settings. This could influence the management of radiation-induced conditions like osteoradionecrosis, fibrosis and cancer metastasis. Overall, our study underscores the need for further research into the effects of radiation on structural proteins to better understand and mitigate radiation-induced tissue damage.

## Introduction

Ionizing radiation is electromagnetic energy capable of ionizing atoms or molecules by removing an electron. This includes photons with wavelengths less than 200 nm, such as far ultraviolet, x-rays, and γ-rays[1,2]. Clinically, ionizing radiation is used in diagnostic radiology, radiation therapy, and sterilization of implantable tissues like skin grafts [3]. Dose levels vary: diagnostic doses are in the µ Gy to mGy range, therapeutic doses in the Gy range, and sterilization doses in the kGy range [1]. The interaction of ionizing radiation with materials depends on photon energy and the chemical composition of the absorbing material. High-energy interactions, such as those in radiotherapy or sterilization, predominantly involve Compton interactions with outer electrons and pair production mechanisms, causing biological damage. Lower energy interactions in diagnostic imaging mainly involve the photoelectric effect, influenced by the atomic number of the absorbing material l[4,5]. Biological damage from radiation can be direct or indirect. Direct damage involves direct ionization of molecules, while indirect damage involves the formation of free radicals, especially in interactions between x-rays and water, leading to ionized water and hydroxyl radicals [6–9]. The actual cell death and tumour eradication is caused primarily by cellular DNA damage (double strand breaks) in conventional radiotherapy[10] although microvascular damage may also play a role at higher-dose-per-fraction clinical regimens. [11, 12].The radiation effects on connective tissue, particularly collagen, have been less studied compared to cellular cytotoxicity. While high doses used in sterilization (50-1,000 kGy) are known to cause fragmentation and crosslinking in collagen, the impact of lower doses used in diagnostics and therapy on collagen structure and properties remains relatively underexplored [13–19]

Hydration levels of tissues, influenced by various physiological and environmental factors, are known to modulate the effects of radiation on collagen. Studies show that both dry and wet irradiation conditions can significantly alter resulting collagen properties, including solubility, thermal capacity, and tensile strength [14–16,20–22]. While high-dose studies have demonstrated collagen degradation and fibril reorganization, the effects of clinically relevant doses (tens of Gy) on collagen’s mechanical properties, especially at the fibril level, have not been thoroughly assessed [19,23]

This study addresses the knowledge gap by investigating the impact of clinically relevant and sterilization dose levels of ionizing radiation on collagen’s morphometric and functional properties under different hydration conditions. These insights could lead to novel therapeutics and modifications of current treatments to minimize adverse effects on healthy tissues.

## Methods

### Collagen scaffolds engineering

Acellular collagen scaffolds were prepared using a protocol modified from Brown et al. [1]. Briefly, 5 mL of rat-tail single molecule type I collagen solution (2 mg/mL in 0.6% acetic acid; First Link Ltd., West Midlands, U.K.) was homogenously mixed with 0.5 mL of 10x and 0.5 mL of 1x Minimum Essential Medium Eagle (Millipore Sigma, St. Louis, MO, USA) to yield a final concentration of 1.67 mg/mL of collagen. The solution was neutralized dropwise to physiologic pH (~7.4) using 5M and 1M sodium hydroxide (NaOH). The neutralized solution was then transferred into the wells of a 24-well plate with 1 mL of solution per well and incubated for 30 min at 37°C in a humidified incubator to allow fibrillogenesis. After incubation, scaffolds were held at room temperature for 5 minutes before rinsing with phosphate buffer saline (PBS) solution thrice. The scaffolds were then plastically compressed following a protocol described by Brown et al. [1] Scaffolds were rinsed with PBS thrice before being segmented using a 5 mm biopsy punch to form the final collagen samples. Two samples were paired to match irradiated samples with their corresponding non-irradiated controls obtained from the same collagen scaffold to minimize engineered collagen variability and enable meaningful comparisons of radiation effects. These paired samples were always kept in the same storage conditions, with only the irradiation procedure being different. Fig 2figa. represents a graphical summary of the sample preparation procedure.

**Fig 1:**
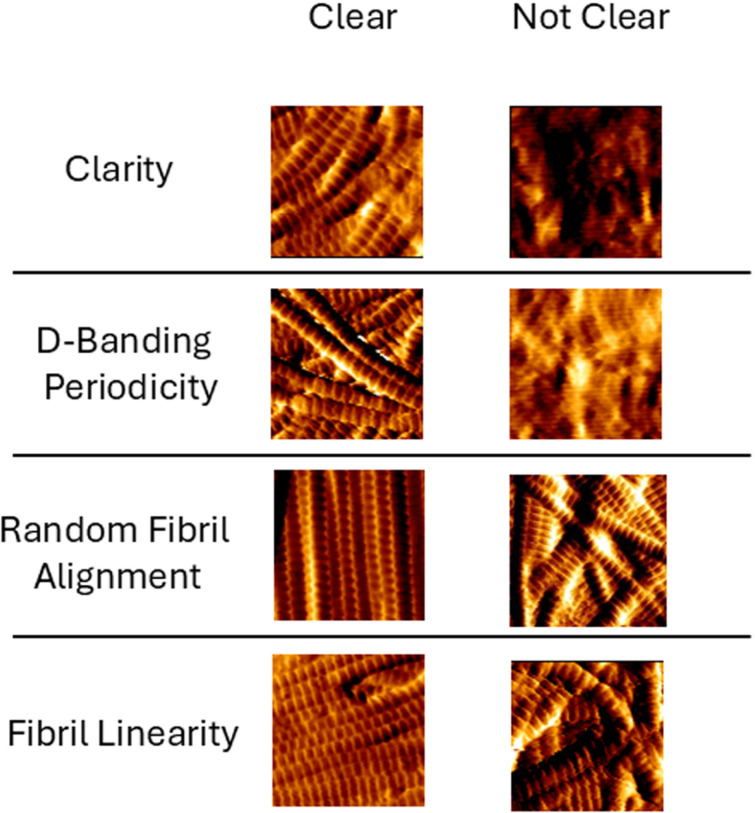
Collagen morphometric qualitative assessment classifier. Each 1 µm ×  1 µm AFM image represents one of the four classifiers: clarity, D-banding periodicity, random orientation of fibrils, and linearity of fibrils.

### Irradiation of collagen samples

To explore the impact of sample hydration on the irradiation of collagen samples, all pairs of collagen samples were randomly assigned to either the dry or the wet state irradiation group. Samples from the dry-state irradiation group were first rinsed with de-ionized (DI) water thrice for 10 min before being mounted onto a glass substrate and physisorbed for 24 hours before irradiation. The mounted samples were then placed into the irradiation chamber in this air-dried state. For the wet state group, samples were placed into a PBS-filled Falcon tube (Thermo Fischer Scientific, Waltham, MA, USA) for at least 24 hours before irradiation to ensure complete hydration. The sample was kept inside the PBS-filled container and placed into the irradiation sources as described below. Three biological replicates were prepared for each irradiation and hydration group, and each sample was irradiated identically in an entirely separate experiment.

For diagnostic dose-level irradiation, samples (3 dry and 3 wet samples) were centrally positioned onto a table-top mount in a Veraview X800 cone beam CT unit (J. Morita Corp., Kyoto, Japan) and irradiated at 100 kV and 5 mA, with 360 degrees of acquisition. The estimated dose from this protocol is 50 µ Gy, similar to other cone beam CT units using a similar field-of-view size, kVp, and mA [2]. In this setup, the energy of a single photon is 1.602 × 10^ − 14^ J with a wavelength of λ_diag_ = 0.0124nm.

For therapeutic dose-level irradiation, samples (3 dry and 3 wet samples) were irradiated using an X-RAD 225Cx small animal irradiator (Precision X-ray, North Branford, CT, USA) through a 10 mm diameter collimator at 225 kVp, 13 mA, and with a 0.3 mm copper filter. The dose rate was approximately 2.65 Gy/min at the isocentre, to a total irradiation dose of 70 Gy in a single fraction. The system was calibrated following the American Association of Physicists in Medicine TG-61 protocol. In this setup, the energy of a single photon is 3.6045 × 10^ − 14^ J with a wavelength of λ_thera_ = 0.0055nm.

For sterilization dose-level irradiation, samples (3 dry and 3 wet samples) were irradiated using a Gamma cell 220 Cobalt-60 unit (Nordion, Ottawa, Canada) at 0.3 kGy/hour to 25 kGy over 3.5 days. In this setup, the energy of the photons is determined by the gamma rays emitted from Cobalt-60. Cobalt-60 emits gamma rays with two primary energy peaks at approximately 1.17 MeV (or 1.87434 × 10^ − 13^J) and 1.33 MeV (2.12966 × 10^ − 13^J) with the corresponding wavelength of λ_Cob1_ = 0.00106nm and λ_Cob2_ = 0.00093nm. Note that Plastic, such as polypropylene or polystyrene used in Falcon tubes, can absorb and scatter X-rays, but the effect is relatively minimal at diagnostic energy levels (100 keV). Most of the radiation will still reach the sample. Plastic will still absorb and scatter some radiation at higher energy levels (225 keV), but again, most radiation will penetrate the tube and reach the sample. The higher energy photons are more penetrating. Thus, any absorption of photons by the falcon tube would be considered a systematic error. Since our scaffold does not contain cells, enzymes, or other proteins, it cannot be altered or repaired over time in response to an incumbent radiation dose. Thus, we decided to use a single fraction (the entire radiation dose given in a single session) rather than splitting it over multiple sessions, as described by Miller et al ^3^. Following irradiation, samples were removed from PBS, rinsed three times with DI water, and allowed to be physisorbed onto a glass substrate. A positive control for denaturation was performed by thermally denaturing a sample placed into a PBS-filled Eppendorf using a hot block set at 100°C for 90 min. All samples were stored at 4° C until characterization, performed within one-week post-irradiation. For AFM characterization, the samples from the wet-state irradiation group were first rinsed with de-ionized (DI) water thrice for 10 min before being mounted onto a glass substrate and physisorbed for 24 hours before irradiation.

### Atomic force microscopy (AFM) imaging and indentation

An atomic force microscope (JPK Nano-wizard^®^ 4, Bruker, Manheim, Germany) was used for imaging and indenting the collagen samples. Imaging was performed in contact mode under ambient conditions using a silicon-tipped MSLN-10-C probe (Bruker, Mannheim, Germany). At least four 10 µm by 10 µm images were acquired in the middle of each collagen sample at random sites with optimized gains settings at 1 Hz; over 200 images were acquired in total. Indentation was performed under ambient conditions on individual collagen fibrils using FESPA-V2 probes (Bruker, Mannheim, Germany) with a nominal spring constant of 2.8 N/m. At least 300 indentations were made at no less than the three locations on each sample, leading to a minimum of 900 indentations per sample.

AFM calibration was done according to our previous work[24]. Briefly, after laser alignment on the AFM, the system was calibrated to acquire cantilever sensitivity and spring constant using a clean glass slide. Once these values were acquired, the glass slide was replaced with the sample slide. Initially, a low-resolution image (128 ×  128 px over 10 ×  10 μm2) was performed to ensure that collagen fibrils with defined D-banding periodicity could be observed. Then, indentation sites were manually selected directly on distinct collagen fibrils’ D-banding (overlap region). All indentations were carried out at 1Hz, with a maximum indentation load not exceeding 100 nN (yielding an average indentation depth of d =  10 ±  1 nm). The indentation modulus (or Young’s Modulus) was obtained from all measurements by fitting a Hertzian model to the force-distance curve [25,26]. In all the analysis, the tip shape is set as a quadratic pyramid with the pyramid half angle to face set as 20.8º. All image processing and data analysis was performed using the JPK data processing software (version 6.3.5). An example of a force-distance curve used for the calculation of indentation modulus can be found in supporting information S1 Fig”. indentation modulus distribution for all the experimental groups can be found in supporting information S2 Fig

### Qualitative image analysis

Acquired AFM images were reviewed qualitatively, with the experimental (irradiated) samples compared to their respective controls. This qualitative analysis enabled us to assert which collagen morphological features may be of interest in exploring the impact of radiation on collagen topology. While this approach relies on establishing empirical morphological features, it has proved pertinent in other studies [27,28]. From this qualitative assessment, we propose the following four collagen morphometric classifiers: clarity, D-banding periodicity, random orientation of the fibrils, and linearity of the fibrils. As Shown in Fig 1 clarity is the ability of the viewer to identify clear edges between individual fibrils. In an AFM image presenting clear fibrils, the fibrils in the field of view can be resolved with distinguishable edges observed between them. This feature is essential as fibrils undergoing degradation appear to lose distinguishable edges and merge into an amorphous collagen layer. D-banding periodicity refers to the regular annular periodicity along the long axis of collagen fibrils. This periodicity is highly conserved across all tissue and species in vivo and results from the correct staggering of the collagen molecules during fibrillogenesis. The fibrils should exhibit well-defined and regular D-banding periodicity in an AFM image with regular D-banding periodicity. Random orientation of the fibrils refers to the isotropic fibrils’ arrangement within the engineered collagen scaffolds. In general, native and healthy collagen tissues tend to have a well-characterized fibrillar anisotropy. Linearity of the fibrils refers to the long and straight morphology of individual collagen fibrils over the field-of-view (here 10 µm by 10 µm). Poorly formed or damaged fibrils have a kinked or twisted appearance [29,30].

### Automated collagen scaffold texture analysis

Automated texture analysis with a neural network was used to analyze the AFM images further. Texture classifiers were implemented in MATLAB software (version 2021a; MathWorks, Natick, MA, USA). Each of the acquired 10 µm by 10 µm field of view AFM images (n = 207) were subjected to a ‘patching’ process, by subdividing them into one hundred 1 µm by 1 µm (51 by 51 pixels) regions-of-interest (ROIs). Each area was assessed using the four binary metrics of fibril clarity, D-banding periodicity, random fibril alignment, and fibril linearity. The texture classifiers consisted of the spatial-spectral network (SSN) as the feature extractor for every given ROI, considering a neighborhood of radius 6 pixels classified for each of the four metrics independently, according to linear discriminant analysis (LDA) model ^5^. Supervised training was performed on 30 randomly selected representative AFM images (10x10µm^2^) of the entire dataset consisting of at least one example of each experimental group. One investigator manually labeled the 3,000 ROIs 1x1µm^2^) for all four features. Two other experts then selected random ROIs (~50) to confirm the accuracy of the labeling. Subsequently, the ROIs of 7 of the training set images were repeated three months apart to quantify intra-user variability and determine a target level of consistency for the trained texture classifier. Performance for each of the four metric classifiers was evaluated based on the accuracy of leave-one-out cross-validation (whereby we iteratively cycle through predicting each of the 3000 ROIs of the training set by training the LDA texture classifiers on the remaining 2999 ROIs). Once the classifiers labeled the dataset of images, approximately 300 of the ROIs were randomly sampled by the ground-truth investigator to validate further that the accuracy was as projected by the leave-one-out cross-validation.

### Attenuated total reflectance using Fourier transform infrared spectroscopy (ATR-FTIR)

The infrared spectra of the collagen samples (irradiated & control) were recorded by positioning the scaffolds directly on the diamond window of a GladiATR (Pike Technologies, Madison, WI, USA) mounted inside an iS20 FTIR spectrometer (Thermo-Scientific, Waltham, MA, USA) with 4 cm^-1^ resolution and 32 scans. Spectra were obtained between 800 cm^-1^ and 4000 cm^-1^, and the bands of interest, namely the Amide I, II, and III regions, were analyzed following baseline correction and normalization.

### Time-correlated single-photon counting (TCSPC) Fluorescence Lifetime Imaging Microscopy (FLIM)

TCSPC FLIM was performed on irradiated and control samples with an inverted confocal laser scanning microscope (A1R, Nikon, Japan) with a FLIM add-on module (LSM Upgrade Kit, PicoQuant, Germany) and SymPhoTime 64 software for data acquisition and fitting (PicoQuant, Germany). Fluorescence was excited with a picosecond pulsed 405 nm diode laser operating at a 5 MHz repetition rate[18]. The photons from the decay were collected with a hybrid PMT for 200 ns with 50 ps bin width. To avoid photon pile-up, excitation intensity was adjusted such that the data collection rate did not exceed 1% of the excitation repetition rate. The scaffolds were placed on a #1.5 coverslip and imaged with a 20x NA0.75 objective (Nikon, Japan). A randomly chosen area of 213 µm by 213 µm was scanned with 512 by 512 pixels until the peak of the sum decay had at least 10^6^ photons and decays from each pixel were added together for lifetime analysis. The experiment was repeated for three randomly chosen areas from each sample.

The sum decay for each area was fitted with a three-exponential function:


$$\text{I}\left(\text{t}\right)={\text{α}}_{1}\text{exp}\left(-\frac{\text{t}}{{\text{τ}}_{1}}\right)+{\text{α}}_{2}\text{exp}\left(-\frac{\text{t}}{{\text{τ}}_{2}}\right)+{\text{α}}_{3}\text{exp}\left(-\frac{\text{t}}{{\text{τ}}_{3}}\right)$$ (Equation 1)

where I(t) is the measured fluorescence intensity, α_1_, α_2_ and α_3_ are the relative amplitudes at t = 0, and τ_1_, τ_2_ and τ_3_ are the decay times. In Time-Correlated Single-Photon Counting (TCSPC) Fluorescence Lifetime Imaging Microscopy (FLIM), the decay curve of fluorescence intensity is modeled with three terms to accurately capture the fluorescence decay process’s complexity. Our collagen samples often contain multiple fluorescent species with different lifetimes. A single exponential decay model would be insufficient to describe such a system. We can account for multiple species and their respective lifetimes using three terms, providing a more accurate representation of the fluorescence decay. In addition, the fluorescence decay is influenced by radiative processes and non-radiative processes such as quenching, energy transfer, and interactions with other molecules. These processes can create complex decay patterns that are best described by multiple exponential terms.

Therefore, the average fluorescence lifetime was determined from [31]:


$$\bar{\text{τ}}=\frac{{\text{α}}_{1}{\text{τ}}_{1}+{\text{α}}_{2}{\text{τ}}_{2}+{\text{α}}_{3}{\text{τ}}_{3}}{{\text{α}}_{1}+{\text{α}}_{2}+{\text{α}}_{3}}$$ (Equation 2)

The average lifetimes were then calculated by averaging all measurements for each treatment condition (i.e., 3 measurements per sample, 3 to 6 samples per treatment condition) and errors were obtained from the standard deviation.

### Statistical analysis

Prism (GraphPad Software, San Diego, CA, USA) was used for statistical testing. The Mann-Whitney U test was used to compare controls to their respective irradiated samples, whereas Kruskal-Wallis ANOVA and Dunn’s multiple comparison tests were used for multiple comparisons. Statistical significance was set at p < 0.05.

## Results

### In vitro engineering of collagen

**Fig 2:**
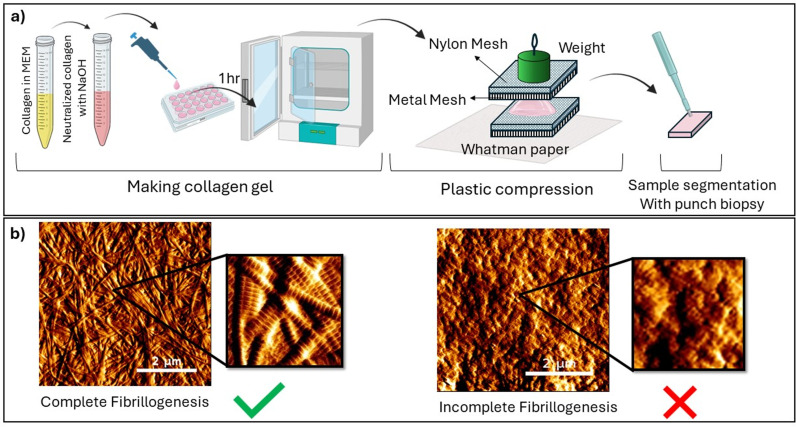
(a). Graphical summary of the sample preparation procedure. (b). Representative AFM images. Left: Collagen fibrils having undergone complete fibrillogenesis and displaying a well-defined fibrillar morphology with clear edges and D-banding periodicity. Right: Collagen fibrils having undergone an incomplete fibrillogenesis and exhibiting a non-fibrillar morphology (amorphous collagen) with no observable D-banding periodicity.

While in vitro engineering of collagen samples has been widely used for tissue engineering applications, very few studies have investigated how well-formed the collagen fibrils were engineered with this protocol. Fig 2b shows two representative topological AFM images of collagen samples obtained from the same protocol, yet some critical variations in the collagen fibril morphology are evident. In Fig 2b. (left image), the fibrils can be resolved with clear edges observed between individual fibrils. The D-banding periodicity is also visible along the long axis of the fibrils. The randomly oriented fibrils present a uniform, long, straight morphology over the field of view. This is directly contrasted with Fig 2b. (right image) where the collagen fibrils are not resolvable. Distinct and linear fibrils cannot be discerned, and no D-banding periodicity is visible throughout the field of view. The lack of distinct collagen fibril morphological features (as seen in Fig 2b. left image) suggests that fibrillogenesis of the collagen fibrils was incomplete, likely because of inadequate pH neutralization, resulting in an amorphous collagen scaffold rather than a scaffold presenting well-structured collagen fibrils [26,32,33]. Unfortunately, the literature has never discussed this issue of incomplete fibrillogenesis. This can only be observed by performing a quality control of the collagen fibrils formed at the nanoscale, where the collagen D-banding can be readily observed [34], which may explain the paucity of related reports. Therefore, in our study, all collagen samples were quality-checked to ensure that all pre-radiation samples presented similar collagen fibrils morphology defined by the clarity, D-banding periodicity, random orientation of the fibrils, and linearity metrics discussed above.

### Impact of irradiation on the morphology of collagen fibrils

#### Morphometric (qualitative) assessment.

**Fig 3:**
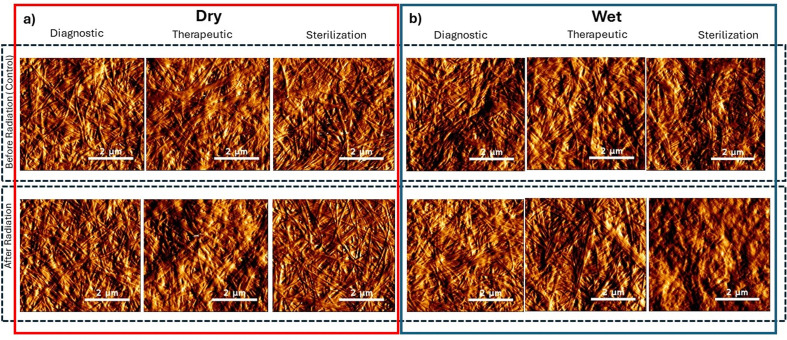
Representative topological images (5x5µm) of collagen samples irradiated in the dry state (a). and wet state (b). All collagen fibrils conserved their well-defined morphology (fibril clarity, visible interfibrillar edges, observable D-banding periodicity, random fibrillar orientation and linearity) across all radiation doses and hydration status, except for the fibrils from the sterilization dose level in the wet, which present compromised morphologies.

It has been reported that collagen fibrils’ morphology and arrangement can be altered following exposure to sterilization levels of ionizing radiation in ex vivo tissues and at therapeutic dose levels in vivo[13,35–38]. In ex vivo tissues, the hydration status at the time of irradiation has been shown to result in different patterns of morphological change. We first explored the effects of ionizing radiation on the qualitative appearance of the collagen fibrils, focusing on the previously described features of fibril clarity, D-banding periodicity, random fibril orientation, and fibril linearity. Fig 3a. shows representative topological images of collagen samples of the experimental groups irradiated in the dry state obtained within 7 days from the time of irradiation. The delay between the irradiation time and the imaging time did not impact the morphology of the collagen as the sample were physisorbed (air-dried) onto a glass substrate immediately post-irradiation and stored at 4°C. The topological images in Fig 3a show that the control samples display a collagen fibril morphology that cannot be differentiated visually at the diagnostic, therapeutic, and sterilization dose levels. The fibrils in the field-of-view are clear (fibrils visible with defined interfibrillar edges), D-banding periodicity is visible, and the fibrils are straight within the image range and randomly oriented throughout the image. It thus appears impossible to visually identify any subtle variation in the collagen fibril morphology pre- and post-irradiation (in the dry state) regardless of the dose level used. Fig 3b. represent topological images of collagen samples pre- and post-wet irradiation. Analogous to the dry state results, the topological images show that the control and irradiated samples at the diagnostic and therapeutic dose levels also display a collagen fibril morphology that cannot be visually differentiated from one another. The fibrils in the field-of-view are clear (fibrils visible with defined interfibrillar edges), D-banding periodicity is visible, and the fibrils are straight within the image range and randomly oriented. However, in the pre-and post-irradiation images at the sterilization dose level, the collagen fibrils are not clearly visible, D-banding periodicity is only sporadically present, and fibril linearity is lost. Interestingly, these features are seen in both the pre-and post-irradiation samples, which suggests that these changes may not be arising from ionizing radiation, but likely caused by the excessive storage time ( ~ 2 weeks) in media for sterilization dose-level group that impacts the stability of the collagen fibrils that start to unwind (de-fibrillogenesis) [39]. This phenomenon was already observed in a previous study [40]

Overall then, the qualitative assessment shows no visually observable morphological changes between the control and irradiated samples across all investigated groups.

#### Automated quantitative topological assessment of collagen fibrils and scaffolds.

To improve our image analysis, we used an automated neural network to explore any variations in the morphology of the collagen fibrils exposed to the three different radiation doses. We define the metric “consistency” as a measure of accuracy and repeatability which will be used to compare the manual labelling and automatic labelling techniques and validate the utility of using the latter in the place of the former. As described in section “Automated collagen scaffold texture analysis”, our ground truth labelling for each of the four considered binary metrics (clarity, D-banding periodicity, random orientation of the fibrils, and linearity of the fibrils) was manually performed by one investigator across 3,000 ROIs representative of the diverse range of experimental conditions (with at least 100 ROIs for each condition). ~ 50 randomly selected ROIs labelling was confirmed independently by 2 other experts, supporting the accuracy of the ground truth labelling. However, the question of intra-user variability remained in whether the ground truth labelling was being performed repeatability across all ROIs. Therefore, three months after completing the labelling of the 3000 ROIs, 700 arbitrarily picked previously labelled ROIs were relabelled by the same ground-truth investigator, and correspondence between labelling at the separate time points was measured as the “consistency” metric. Next, when considering the automatic classifiers for each of the 4 binary metrics an opposite issue is faced; repeatability is not the concern, but rather the accuracy. Thus, through leave-one-out cross-validation, the accuracy value obtained by comparing with the ground truth labels was used as its “consistency” metric. The results of these investigations are summarized in Table 1. There was greater consistency associated with the neural-network texture classifier for each of the four assessed categories than the manual labeling: the automated classifier was 11%, 25%, 9%, and 23% more consistent than manual labeling. These results suggest the neural network classifier as being more reliable than the “standard” of manual labeling and support its utilization for analysis of the entirety of the AFM image dataset. Following this initial calibration, the classifier was tasked to assess all ROIs generated by the subdivision of the entire AFM images dataset (20,700 ROIs generated from 207 AFM images). The consistency of this assessment is presented in Table 1.

**Table 1:** Comparative summary table of the manual versus neural-network texture classifier labelling consistency for each of the empirical metric classifiers used to characterize individual collagen fibrils from the AFM images. The consistency is expressed in percentage for each approach.

Metric classifier	Manual labelling consistency (%)	Linear discriminate analysis classifier consistency (%)
**Clarity of fibrils**	75	86
**Clarity of D-banding**	59	84
**Linearity of fibrils**	85	94
**Random orientation of fibrils**	70	93

Since the automated labeler is consistent with the ground truth labeling > 80% of the time (86% for fibril clarity, 84% for D-banding periodicity clarity, 94% for fibril linearity, and 93% for random orientation of the fibrils), the probability that the labeler will mislabel an ROI is 1 minus the percentage consistency for each of the four categories (14% for fibril clarity, 16% for D-banding periodicity clarity, 6% for fibril linearity, and 7% for random orientation of the fibrils) as summarized in Table 2. Hence, when comparing results from different samples, only those differences greater than these values (the “threshold value”) should be considered reflective of experimentally observable differences.

**Table 2:** Percentage change in the four assessed criteria between the paired samples (one irradiated – one control – same storage conditions) by the automated labeler by dose level and hydration status relative to the control sample. DD: dry diagnostic, DT: dry therapeutic, DS: dry sterilization, WD: wet diagnostic, WT: wet therapeutic, WS: wet sterilization.

	% Change in D-banding periodicity clarity	% change in fibril clarity	% change in random fibril orientation	% change in fibril linearity
**DD**	0	0	+1	-1
**DT**	+4	-4	+5	-2
**DS**	0	+1	+1	0
**WD**	+9	-2	-2	-5
**WT**	-2	0	+1	+1
**WS**	-16	-28	-2	+9
**Threshold value**	±14%	±16%	±6%	±7%

With the automated classifier pipeline thus optimized, the controls and the irradiated samples were compared to assess radiation-induced effects in the engineered samples as presented in Table 3.

**Table 3:** Automated pixel analysis of collagen samples by dose-level and hydration status for D-banding periodicity clarity, fibril clarity, random orientation of fibrils, and linearity of fibrils. CDD = control dry diagnostic, R.DD = radiation dry diagnostic, CDT = control dry therapeutic, RDT = radiation dry therapeutic, CDS = control dry sterilization, RDS = radiation dry sterilization, CWD =  control wet diagnostic, RWD =  radiation Wet diagnostic, CWT = control \Vet therapeutic, RWT =  radiation Wet therapeutic, CWS =  control wet sterilization, RWS =  radiation wet sterilization. n =  number of pixels.

	D-banding periodicity clear (%)			Fibrils clear (%)			Random fibril orientation (%)			Fibril linearity (%)		
**N events**	**Yes (%)**	**No (%)**	**N events**	**Yes** **(%)**	**No** **(%)**	**N** **events**	**Yes** **(%)**	**No** **(%)**	**N** **events**	**Kinked** **(%)**	**Linear** **(%)**
**CDD**	1800	98	2	1800	97	3	1800	98	2	1800	2	98
**RDD**	1900	98	2	1900	97	3	1900	99	1	1900	1	99
**CDT**	2000	87	13	2000	88	12	2000	93	7	2000	6	94
**RDT**	1700	91	9	1700	84	16	1700	98	2	1700	4	96
**CDS**	1200	81	19	1200	94	6	1200	96	4	1200	3	97
**RDS**	1200	81	19	1200	95	5	1200	97	3	1200	3	97
**CWD**	2500	76	24	2500	77	23	2500	93	7	2500	10	90
**RWD**	2400	85	15	2400	75	25	2400	81	19	2400	5	95
**CWT**	1800	94	6	1800	93	7	1800	93	7	1800	3	97
**R\VT**	1800	92	8	1800	93	7	1800	94	6	1800	4	96
**C\VS**	1200	73	27	1200	69	31	1200	97	3	1200	8	92
**RWS**	1200	57	43	1200	41	59	1200	95	5	1200	17	83

No change was observed in any of the four binary criteria greater than the threshold value for samples irradiated in the dry state. This demonstrates no detectable morphological change to the collagen fibrils induced by ionizing radiation at any dose level and is consistent with our qualitative assessment of the collagen samples. There is similarly no change in the four criteria for diagnostic and therapeutic dose levels for samples irradiated in the wet state. However, an interesting result is seen in the collagen samples irradiated at sterilization dose levels in the wet state. Here, the percentage change in three of the four categories assessed (D-banding periodicity clarity, fibril clarity, and fibril linearity) exceeds that of the threshold values. The irradiated samples show a decrease in D-banding periodicity clarity, a reduction in fibril clarity, and an increase in fibril kinking compared to the control, which all indicate radiation-induced deterioration. Thus, the automated analysis suggests that changes in this sample group occur to the collagen fibrils due to irradiation, storage issues associated with this group notwithstanding (see above). These results show that our automated analysis method can recognize visually unobservable morphological changes in AFM images through qualitative assessment and that despite similar appearance, changes are induced in sterilization dose-level samples when irradiated in the wet state.

#### Impact of irradiation on the mechanical properties of collagen fibrils.

We next explored how indentation modulus of individual collagen fibrils within each collagen sample varied following dry or wet irradiation at diagnostic, therapeutic, and sterilization dose levels using AFM-based indentation performed directly on individual fibrils. Table 4 summarises the median indentation moduli for all the groups. The collagen fibrils indentation moduli average to (4.82 ± 0.52) GPa with a ~ 10% variation across all control groups.

**Table 4:** Comparative summary table of the indentation moduli values (median) as a function of all dose level and hydration status. The variation in the median values as a function of radiation dose received is expressed in %. While all the dose level groups exhibited a variation less than 10% in their indentation moduli values (median) regardless of the hydration status, the sterilization group presented a reduction of 26.4% in its indentation moduli value (green) in the dry condition and increase of 55.8% in its indentation moduli value (orange) in the wet condition. All variations in indentation moduli values (median) as a function of all dose level and hydration status were found to be statistically significant (p ranging from p <  0.05 to p <  0.0001) except for the dry diagnostic group (p >  0.05).

Hydration Status	Dose-level	Group	n	Median Indentation Modulus (GPa)	Variation	Significance	
**Dry**	Diagnostic	Control	900	4.82 (±0.02)	+ 2.1%	No	p > 0.05
		Radiation	900	4.93 (±0.04)			
	Therapeutic	Control	900	4.83 (±0.11)	- 8.3%	Yes	p < 0.005
		Radiation	900	4.42 (±0.12)			
	Sterilization	Control	900	5.43 (±0.1)	- 26.4%	Yes	p < 0.0001
		Radiation	900	3.92 (±0.15)			
**Wet**	Diagnostic	Control	900	3.94 (±0.14)	+7.7%	Yes	p < 0.0001
		Radiation	900	4.21 (±0.11)			
	Therapeutic	Control	900	4.65 (±0.06)	+6.5%	Yes	p < 0.05
		Radiation	900	4.93 (±0.07)			
	Sterilization	Control	900	5.27 (±0.15)	+55.8%	Yes	p < 0.0001
			Radiation	900	8.10 (±0.18)		

These values are within range of the expected value for an engineering collagen scaffold, and the variations in the median indentation moduli values can be attributed to variations in the engineering of the collagen, level of plastic compression of the sample, and fibrillogenesis completion [18]. No significant difference (p > 0.05) exists in the median indentation modulus following irradiation for samples irradiated in the dry state at the diagnostic dose level. For the dry therapeutic and sterilization dose levels, there is a statistically significant decrease (softening) in the median indentation modulus (p < 0.005 and p < 0.0001, respectively). Given the identified heterogeneity of the engineered collagen scaffolds, a meaningful comparison is made only for the percentage change from each of the experimental groups’ controls. For the therapeutic dose level, there is a decrease in the median indentation modulus of 8%; for the sterilization dose level, there is a decrease in the median indentation modulus of 26%. The decrease for both these groups suggests that the collagen fibrils are becoming less stiff cross-sectionally upon irradiation in a dose-level-dependent manner. Such a decrease in indentation modulus may result from a decrease in the fibril density, which could result from fibril unwinding and/or peptide scission caused by radiation exposure. Since the collagen fibrils are stabilized hydrogen bonds and Van der Waals forces[41] that can be disrupted by ionizing radiation, it is unsurprising that weakened collagen fibrils with altered stability are found post-irradiation. These fibrils were exposed to a cumulative 25,000 Gy dose over 3,5 days which suggest that overall, the impact of such high-radiation doses is very modest on the mechanics properties of these fibrils. Finally, samples from the wet irradiation group demonstrated a statistically significant increase (stiffening) in the median indentation modulus at all dose levels (p < 0.0001 for diagnostic dose level and sterilization dose level, p < 0.05 for therapeutic dose levels). The percentage increase of the median indentation modulus for diagnostic, therapeutic, and sterilization dose levels was 8%, 7%, and 53%. This increase in moduli suggests that the collagen fibrils are becoming stiffer or denser. For both the diagnostic and therapeutic doses, the variation in indentation moduli is independent of the dose level. This is a significant result as it affirms that type I collagen fibrils do not respond mechanically to an increase in radiation doses. However, the variation in indentation moduli at sterilization dose level is reflective of a major modification in the mechanism modulating the collagen fibrils their mechanical properties. In a recent study, Yakimov et al. have shown that gamma sterilization of decellularized bovine pericardium induced more pronounced changes in the mechanical properties of the tissue (also measured by AFM) than in the collagen fibril morphology, corroborating our current findings[42]. As discussed in the introduction, the interaction between the X-ray and water can produce ionized water, free radicals (i.e., hydroxyl radicals), and electrons. The presence of free radicals in collagen has previously been linked to the formation of intermolecular crosslinks, which would impact the mechanical properties of the fibrils [13,43] and is a likely explanation for the observed stiffening of the wet samples by irradiation. These results also suggest that the mechanism modulating the collagen fibrils their mechanical properties can be affected by radiation dose, with both the diagnostic and therapeutic dose levels having limited impact when compared to therapeutic dose level.

### Collagen protein stability as a function of irradiation

**Fig 4:**
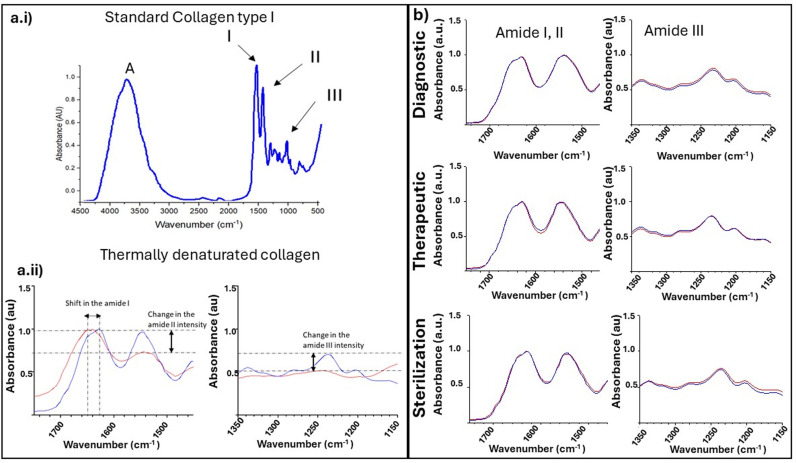
Attenuated Total Reflectance (ATR) Fourier Transform Infrared spectra of (a.i), standard thermally denatured (a.ii), and pre- & post- irradiation (b) Type I collagen samples. All pre-treatment (irradiation or denaturation) spectra are displayed in blue, whereas all post treatment spectra are displayed in red. The standard collagen (a.i) displays the characteristic bands for collagen: Amide A, I, II and III. Upon thermal denaturation, the ratio of the Amide I to II is grossly affected (a.ii) and the Amide III band completely disappear (a.ii). (b) No variations in the Amide I/II band ratio or Amide III disappearance were found as a function of dose level and hydration status.

The impact of radiation on the stability of collagen as a structural protein was investigated using the attenuated total reflectance Fourier transform infrared (ATR-FTIR) technique. The collagen infrared fingerprint comprises distinct absorption bands known as Amide A, I, II, and III. The Amide A band, spanning from 3600 to 3200 cm^ − 1^, corresponds to the stretching vibrations of N–H bonds. Amide bonds within the collagen significantly influence the infrared spectrum, manifested through stretching and bending vibrations. These vibrations include the carbonyl (C = O) stretching at 1650 cm^ − 1^ (amide I band), N–H and C–N bonds at 1550 cm^ − 1^ (amide II band), and 1230 cm^ − 1^ (amide III band) as shown in Fig 4 a.i, a.ii [44].

Fig 4. b represents the infrared spectra of dry irradiated samples compared to their respective controls. In the dry state, at all dose levels (n = 3 for clinically relevant dose levels; n = 6 for sterilization dose level), there is no change in the Amide I (~1630 cm^-1^) or Amide II (~1540 cm^-1^) bands. As both the Amide I and Amide II bands’ position and amplitude do not change as a function of the radiation, this suggests no alteration to the collagen protein confirmation. In the Amide III region of the spectra, the native “triple peak” appearance, with peaks at ~ 1205 cm-1, ~ 1235 cm-1, and ~ 1280 cm-1, is preserved at all dose levels following irradiation in the dry state. Thus, the stability of the Amide III region following irradiation in the dry state at all dose levels further provides evidence for the stability of the collagen molecule. There were no statistically significant differences in the mean Amide I to Amide II ratios (p > 0.05) when comparing all irradiated samples and their respective controls, regardless of the dose levels used or hydration status during the irradiation. This finding corroborates the results from Miller et al. [23] who affirmed that radiation had not denatured the collagen in a similar study.

As a positive control, the ATR-FTIR spectra of thermally denatured collagen (n = 3) were also recorded (Fig 4a.ii). We recorded a visible shift in the wavenumber of the Amide I peak (from ~ 1630 cm-1 to ~ 1640 cm-1), a net reduction in the Amide II band intensity, and a complete loss of the Amide III triplet because of this thermal denaturation. Upon denaturation, collagen fibrils undergo several conformational changes caused by the breaking of different cross-links present at the intermolecular level, such as the nonenzymatic glycosylation of lysine and hydroxylysine residues, and at the intramolecular level, such as the disulfide bridges [34]. Furthermore, the H-bonded water used to stabilize the collagen molecule is released[33], leading to the collapse of the triple helix structure of the molecules. The result of the thermal denaturation of collagen is random fragmentation of the collagen fibril and molecule due to the loss of those cross-links necessary to stabilize the collagen fibril ultrastructure. [45].

Since the infrared spectra of the irradiated collagen did not present any features similar to those observed in the thermally denatured collagen, we conclude that radiation at the examined dose levels does not impact the chemical stability of the collagen molecules. For comparison, the energy absorbed by the sample (mass of 8.9mg) is 0.623 J in the case of the irradiated sterilization group (3 kGy/hour to 25 kGy). In contrast, the energy given to the sample when thermally denatured at 90°C (specific heat capacity of collagen (approximately (3.8 x 10^3^ J/(kg·°C)), is approximately 2.2 KJ. Therefore, there are several orders of magnitude differentials in the energy imparted to the collagen sample when comparing thermal degradation to even the high dose of radiation exposure, which would explain why the collagen remains chemically stable even at the highest radiation dose exposure.

### Fluorescence lifetime characterization of the irradiated collagen

**Fig 5:**
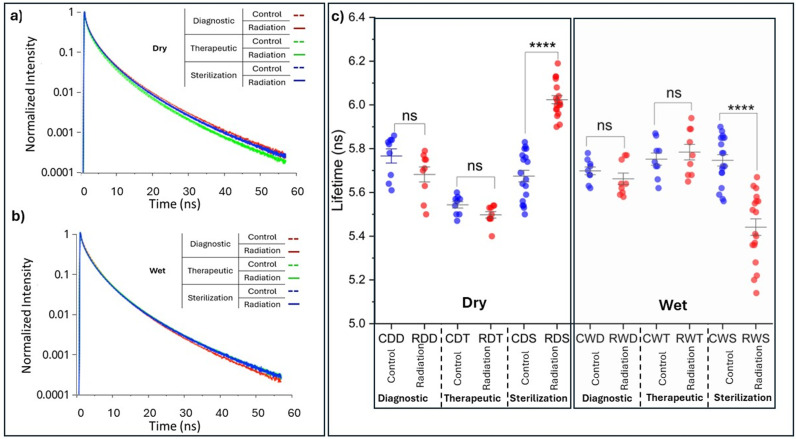
Time-correlated single-photon counting (TCSPC) Fluorescence Lifetime Imaging Microscopy (FLIM) decay curves for all dose level groups as function of their hydration status (a, b) and graphical summary (c) of the variation in the average lifetimes calculated for each treatment condition. In (c), all pre-treatment (irradiation) average lifetimes are displayed in blue, whereas all post treatment average lifetimes are displayed in red. **** denotes statistical significance with p < 0.05.

We performed fluorescence lifetime characterization to explore the possible formation of cross-links within our collagen fibrils due to irradiation. The fluorescence lifetime is an intrinsic property of a fluorophore and is not influenced by fluorophore concentration. Collagen fluorescence originates primarily from crosslinks, and therefore, changes in the intermolecular crosslinks are responsible for variations in the observed fluorescence lifetimes. The fluorescence in collagen is primarily due to specific cross-links and amino acids within the collagen structure. Collagen contains cross-links such as hydroxylysyl pyridinoline and lysyl pyridinoline, which are known to fluoresce when excited by specific wavelengths of light. Certain amino acids in collagen, like tryptophan, tyrosine, and phenylalanine, can also contribute to its autofluorescence. Representative fluorescence lifetime decay curves are shown for dry and wet irradiation conditions (Fig 5 a,b), along with corresponding un-irradiated controls. Some variability is seen in the decay rate of the control samples. This difference may have arisen from variations in the pH during the engineering of the collagen scaffolds, which makes meaningful the comparison between the irradiated samples and their respective controls. The average fluorescence lifetime of the collagen samples was calculated using Equation 2 and is presented in Fig. 5c. At clinically relevant dose levels (i.e., diagnostic and therapeutic dose levels), irrespective of hydration status at the time of irradiation, the fluorescence lifetime values of irradiated samples were not statistically different from those of their respective controls (p > 0.05). This suggests that the crosslinking profiles of the control and irradiated samples remain similar. However, the sterilization dose-level irradiation in both the dry and wet states yielded statistically significant (p < 0.0001) differences in fluorescence lifetime compared to their control. Here, we report an increase of 6% when irradiated in the dry state (5.67 ±  0.11 ns versus 6.02 ±  0.08 ns) and a decrease of 5% when irradiated in the wet state (5.75 ±  0.11 ns versus 5.44 ±  0.16 ns). In our experiments, the amino acid composition of our collagen scaffold does not change as a function of the radiation and, therefore, cannot be the reason for the change in the lifetime. Any variations (increase or reduction) in the fluorescence lifetime of collagen can be explained by the formation of different fluorescent species, for example in the presence of enzymes or glycation agents [15,46]. The sources of the new fluorophores are intriguing as we have not added anything to our collagen scaffold. Yet, our collagen stock was initially made of rat-tail tendons treated in acetic acid. The acid breaks all the intermolecular crosslinks to return the tendons into a single molecule form. Yet, after this process, the collagen molecules may still possess side chains that consist of fragmented crosslinks. We hypothesize whether these fragments of crosslinks could react with radiation to reform said crosslinks or variants. In the absence of any external source of crosslink initiators, the interaction of radiation-induced free-radical with collagen has been suggested to be responsible for the formation of intermolecular crosslinks through a mechanism proposed by Zigler [47] who suggested that single reactive oxygens form hydrogen peroxide (H_2_O_2_) and free radicals (e.g., superoxide anion O^2−^) enable the formation of indirect covalent crosslinks by oxidizing the collagen molecule. Since the fluorescence lifetime is an intrinsic property of a fluorophore and is not influenced by fluorophore concentration, the reduction in the fluorescence lifetime corroborate the formation of different fluorescent species. The recorded decrease in lifetime values demonstrates that new crosslink species formed are AGE-derived as we presented previously[31] and originally proposed by Fukushima [46]. While identifying the newly formed crosslinking species is non-trivial, it is evident that irradiation of the collagen exposed to a sterilization dose level changes the fluorescence lifetime of the collagen, inferring that the high radiation dose does alter collagen crosslinking composition. This result confirms our previous mechanical result for which we suggested that the mechanism modulating the collagen fibrils their mechanical properties is affected by radiation dose especially at therapeutic dose level. Here the mechanism modulating the collagen fibrils’ mechanical and fluorescence lifetime properties is the formation of intermolecular crosslinks in between the collagen molecules essential for fibrillar stabilisation.

## Discussion

Our findings suggest that collagen fibrils are morphologically similar pre- and post-irradiation at all tested dose levels, irrespective of hydration status at the irradiation time. Dose levels higher than clinical and sterilization dose are needed to demonstrate fibril alterations like swelling, D-banding changes, and unwinding. [16,17,48]. Interestingly, unlike other studies, we could not detect changes at doses except for the therapeutic dose level in the organization of the collagen fibrils^18^. This is likely related to our model using randomly oriented engineered collagen fibrils in our scaffolds. In contrast, other models that could detect alterations in organization used native aligned in vivo collagen fibrils. Indeed, other studies using scaffolds at clinical dose levels showed no collagen microarchitecture alterations.[23]. These findings suggest that collagen perturbations at clinical dose levels may result from the inflammatory process following irradiation, rather than inherent fibril alterations[19,49]. This is supported by the absence of immediate alterations in vivo, suggesting they are not directly caused by irradiation on collagen. The radiation did not alter the collagen morphological properties at clinically relevant doses. These findings are consistent with the literature and assessed with AFM and scanning and transmission electron microscopy [19,49]. However, at therapeutic dose levels, collagen fibril properties are affected due to altered mechanisms modulating their mechanics and fluorescence lifetime via intermolecular crosslink formation.

The variations in Young’s moduli of the collagen fibrils were detected in most experimental groups, prominently in the therapeutic one. These variations (i.e., stiffening or softening of the fibrils) are associated with the hydration status of the fibrils at the time of irradiation. These findings are consistent with the literature [17,48,50], previously described at therapeutic and sterilization doses. Still, to our knowledge, we are the first to report on these changes occurring following diagnostic dose levels of irradiation (wet conditions). Two mechanisms may occur with irradiation in the dry vs. wet state, namely peptide scission and crosslink formation [48,50]. Our findings of softening with dry irradiation and stiffening with wet irradiation support this hypothesis, with no clear dose-response between diagnostic and therapeutic doses in the hydrated state. This suggests that insufficient energy may have been imparted onto the sample to induce any alteration in the mechanism modulating collagen fibrils’ mechanical and fluorescence lifetime properties. Our findings align with the literature, showing collagen stability to ionizing radiation across tested doses, with no denaturation at clinical levels and chemical changes only at very high doses[17,23]. These results also support our findings of mechanical changes. They suggest that the mechanical changes detected in the collagen fibrils mechanistically occur without altering the protein conformation. This indicates that changes in the mechanical properties are related to inter- as opposed to intra-protein changes. The infrared spectroscopy results demonstrate the stability of the collagen protein to ionizing radiation in both wet and dry irradiation conditions. Here, we demonstrated the major structural protein in vertebrates remains chemically unaffected at all radiation doses used in this study. This resistance to radiation damage of collagen as a structural protein is significant when considering the impact of radiation on tissues, and we can conclude that high-dose ionizing radiation can induce an inter- as opposed to intra-protein change. The stability of the collagen protein within the fibrils is often associated with the formation of intermolecular crosslinks that stabilize the triple helix. It has been shown that differing intermolecular changes to collagen result in changes in the average fluorescence lifetime. These changes can be of the same type (e.g., cross-linking) but with different directions of change. Thus, the detectable difference in fluorescence lifetime post-irradiation at sterilization dose levels in the wet and dry states suggests alterations to pre-existing fluorophores or the generation of new fluorophores. In our scaffold, there are no pre-existing crosslinks between the molecules. Therefore, we can assert that the change in fluorescence lifetime is directly associated with the generation of new fluorophores. These fluorophores are evidently crosslinks. Furthermore, the direction of change varying between irradiation in the wet state and irradiation in the dry state synergizes with and supports the indentation modulus. The opposing changes in mechanical properties and fluorescence lifetime with hydration support this hypothesis.. It suggests alternate mechanisms by which ionizing radiation interacts with collagen fibrils, mediated by water. Interestingly, we could not detect any changes at diagnostic and therapeutic dose levels in either hydration state with FLIM, though mechanical changes were detected. The 5–6% change at sterilization doses (25 kGy) suggests induced changes may be undetectable due to the 1,000-fold and 100,000-fold dose increases compared to therapeutic and diagnostic levels, respectively. Alternatively, the energy imparted to the tissue at these lower dose levels, irrespective of hydration status at the time of irradiation, may be insufficient to interact with fluorophores or change the microenvironment so as to be detectable by this technique. It is also worth recalling the results from our experimental work with ATR-FTIR, where no changes in the spectra were identified. Therefore, experimental findings from FLIM support the hypothesis that the mechanism modulating the collagen fibrils’ mechanical and fluorescence lifetime properties at high dose levels is the formation of intermolecular crosslinks.

### Clinical relevance of our findings

Most previous radiation biology research has focused on ionizing radiation’s effects on DNA. Our study demonstrates that ionizing radiation can also induce changes in structural proteins, impacting tissues in diagnostic imaging, therapeutic radiation, or sterilization. This expands the understanding of radiation effects on proteins and extracellular matrices. In patient care, particularly radiation therapy, significant undesirable outcomes include osteoradionecrosis, radiation fibrosis and potential extracellular matrix changes facilitating tumor spread. Osteoradionecrosis of the mandible affects 2% to 22% of patients undergoing head and neck radiotherapy, leading to bone necrosis due to reduced vascularity and subsequent vascular insufficiency [51]. Fibrosis, a late effect of irradiation [52] involves abnormal endothelial cell proliferation, muscle cell replacement with collagen fibers, and loss of vessel elasticity. This damage occurs at doses of 50-70 Gy for arteries and 40 Gy for capillaries [10,51]. Since type I collagen is abundant in the extracellular matrix of vessel walls [53,54], irradiation-induced changes in collagen could alter vascular elasticity, contributing to disease development. The extracellular matrix, present throughout the body, influences tumor spread through changes in its mechanical properties [55,56]. A study by Pendelton et al. investigated how ionizing radiation affects bone brittleness by exposing mouse lumbar vertebrae to various radiation doses. The results showed that high doses (17,000 and 35,000 Gy) significantly reduced bone strength due to collagen fragmentation, while non-enzymatic collagen crosslinking increased at all doses but did not correlate strongly with mechanical changes. The findings suggest that radiation-induced reductions in bone strength are more likely caused by collagen fragmentation than by increased crosslinking[57]. Our findings indicate that further research is needed to understand the deleterious effects of diagnostic and therapeutic radiation on non-cellular targets. The ALARA principle (As Low As Reasonably Achievable) primarily addresses radiation-related malignancy but should also consider non-cellular radiation injuries. The clinical significance of these changes needs further quantification. Sterilization with ionizing radiation on implantable materials is another critical outcome. Despite preserving fibril architecture and morphology, our study shows that ionizing radiation induces mechanical and biochemical changes in collagen samples. Implantable materials like bone, skin, and corneas are often sterilized using ionizing radiation [58]. While doses up to 25 kGy are reported not to affect tissue allografts’ physical and biological properties [59,60], our findings suggest that 25 kGy can alter collagen’s physical properties at the fibril level. Whether these changes are clinically significant remains to be determined. Manufacturers and clinicians must consider the effects of sterilization on materials, which may influence how the body responds to them or require modifications to maintain desired properties.

## Conclusion

This study has expanded our understanding of how ionizing radiation affects type I collagen at both fibrillar and molecular levels across various doses and hydration conditions. We found that while the primary structure of collagen remains largely intact, its mechanical properties, such as indentation modulus, change significantly based on the hydration state during irradiation. Hydrated fibrils typically stiffen due to new cross-links, whereas dry fibrils soften due to molecular bond disruptions. These biophysical changes are mostly dose-dependent and have significant clinical implications, especially in oncology and radiology, where radiation is widely used. Alterations in collagen’s mechanical properties can influence radiation-induced disorders like osteoradionecrosis and radiofibrosis and affect the biomechanical environment, influencing tumor progression and metastasis. This highlights the need for a strategic approach in radiation therapy that considers impacts on structural proteins, not just DNA and potentially vascular damage. Understanding radiation’s effects on collagen under varying hydration and doses provides new insights into biomaterial sterilization processes. Given collagen’s role in tissue integrity and function, this calls for a reassessment of current sterilization practices, especially those using high radiation doses, to ensure that biophysical properties essential for biomedical implants are preserved. In conclusion, while collagen’s structural integrity seems resilient to ionizing radiation, its mechanical and biochemical properties undergo significant modifications under specific conditions. This suggests a complex interplay between radiation-induced effects and collagen’s biophysical behavior. Our findings pave the way for future research to mitigate undesirable changes in collagen properties in medical and biomaterial applications, ensuring radiation remains safe and effective. Further research is needed to explore these interactions in vivo and develop strategies to prevent or reverse radiation’s adverse effects on connective tissues, improving patient outcomes and expanding radiation’s utility in medical science.

## Supporting information

S1 Fig An example of a source distant curve, showing Extend (dark blue), retract (light blue) and fitted (green) curves. (TIF)

S2 Fig Indentation modulus of collagen samples irradiated at diagnostic, therapeutic, and sterilization dose levels under wet and dry hydration statuses. Each dot represents one indentation. Bars represent the interquartile range and the median value. CDD =  control dry diagnostic, RDD =  radiation dry diagnostic, CDT =  control dry therapeutic, RDT =  radiation dry therapeutic, CDS =  control dry sterilization, RDS =  radiation dry sterilization, CWD =  control wet diagnostic, RWD =  radiation wet diagnostic, CWT =  control wet therapeutic, RWT =  radiation wet therapeutic, CWS =  control wet sterilization, RWS =  radiation wet sterilization. Statistical significance is indicated as follows: ns =  not significant, *  =  p <  0.05, ** =  p <  0.01, *** =  p <  0.001.
(TIF)

S1 File MATLAB code used for the Automatic characterization of irradiated collagen samples. With the manual on using the code, and the raw data fed to the code.
(ZIP)
