# Supplementary figures and images for: Effects of Ionizing Radiation on the Biophysical Properties of Type I Collagen Fibrils
Source: PLoS One. 2025 Apr 2;20(4):e0319777. doi: 10.1371/journal.pone.0319777 (PMC11964255; doi:10.1371/journal.pone.0319777)

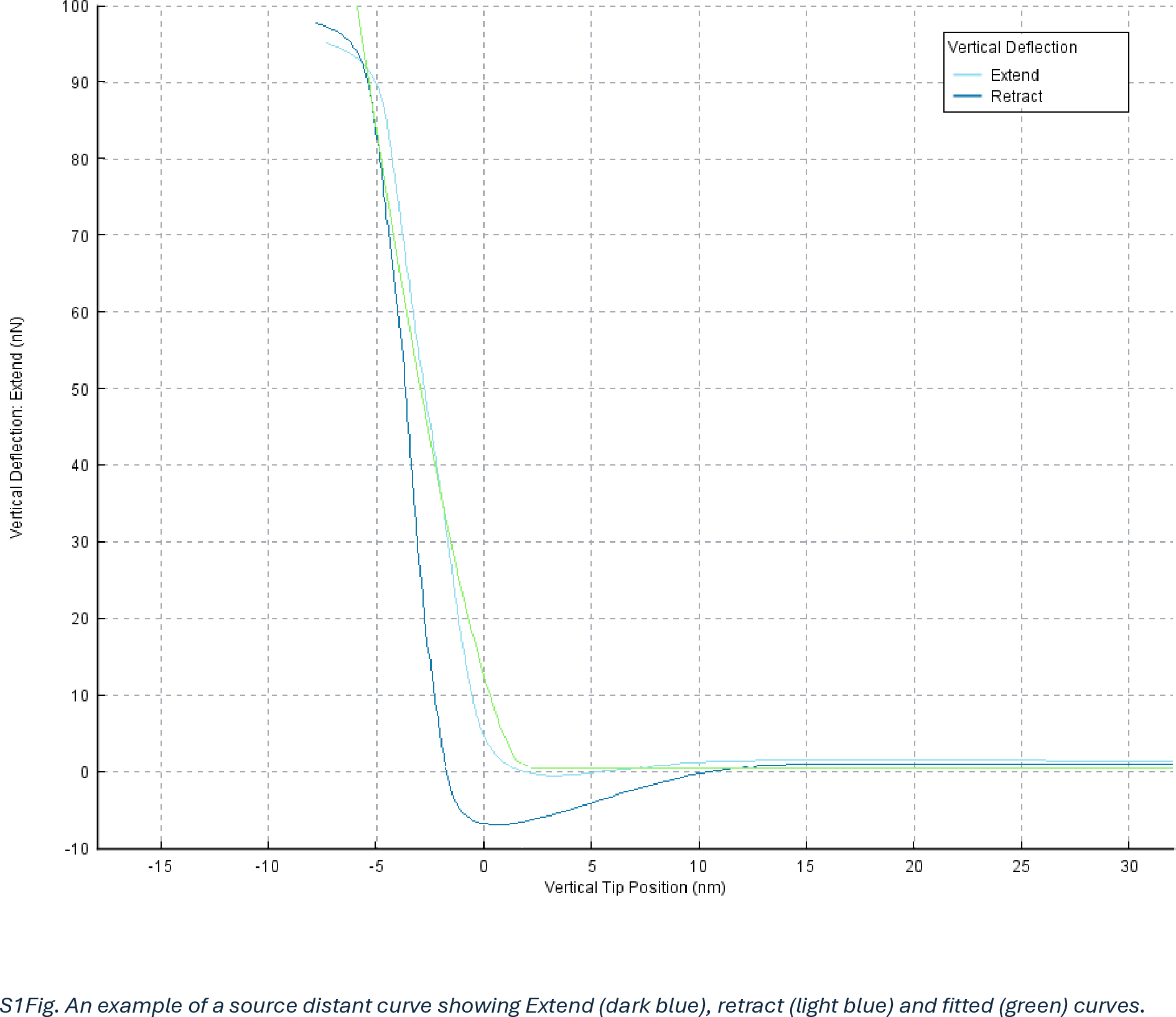

Supplement: S1 Fig — (TIF) [file pone.0319777.s001.tif]

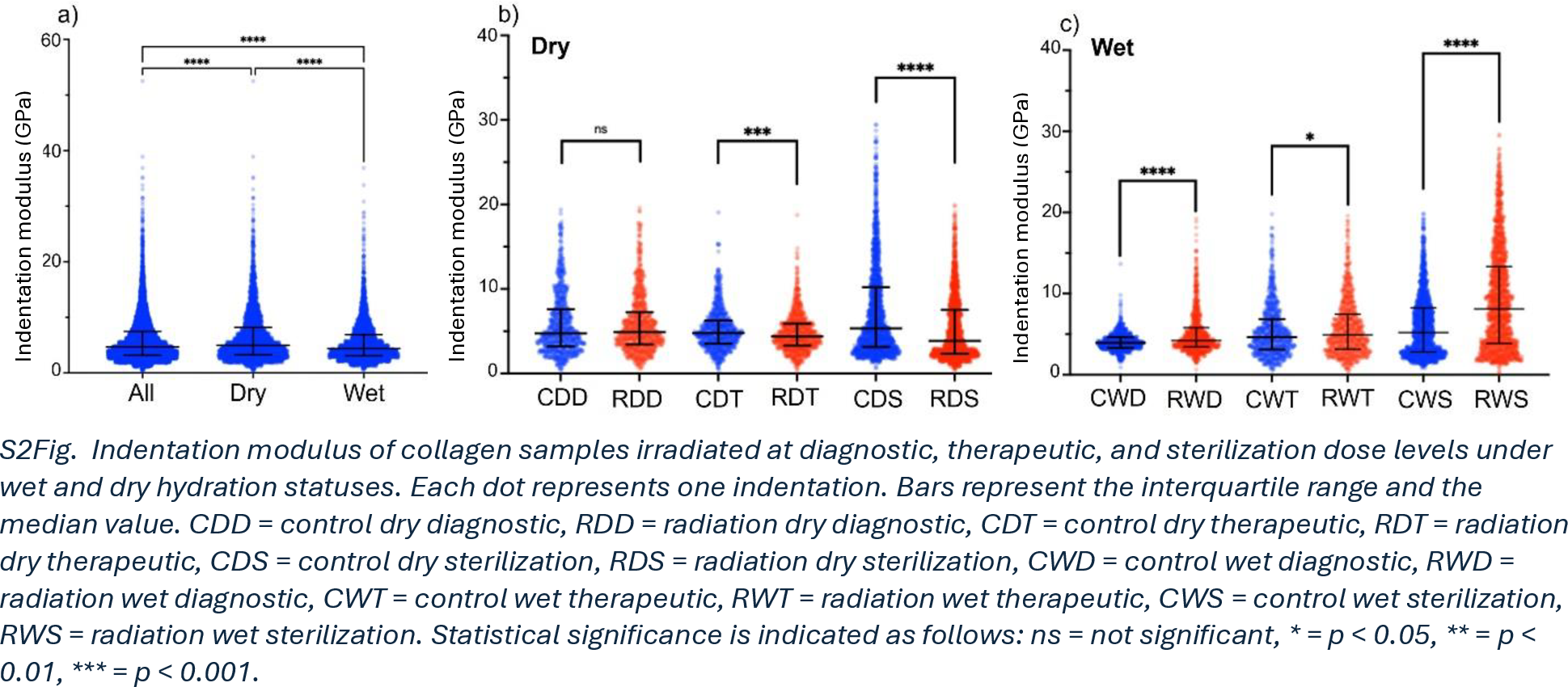

Supplement: S2 Fig — Each dot represents one indentation. Bars represent the interquartile range and the median value. CDD = control dry diagnostic, RDD = radiation dry diagnostic, CDT = control dry therapeutic, RDT = radiation dry therapeutic, CDS = control dry sterilization, RDS = radiation dry sterilization, CWD = control wet diagnostic, RWD = radiation wet diagnostic, CWT = control wet therapeutic, RWT = radiation wet therapeutic, CWS = control wet sterilization, RWS = radiation wet sterilization. Statistical significance is indicated as follows: ns = not significant, * = p < 0.05, ** = p < 0.01, *** = p < 0.001. (TIF) [file pone.0319777.s002.tif]
